# Supplementary material for: Reactive Oxygen Species-Dependent Activation of EGFR/Akt/p38 Mitogen-Activated Protein Kinase and JNK1/2/FoxO1 and AP-1 Pathways in Human Pulmonary Alveolar Epithelial Cells Leads to Up-Regulation of COX-2/PGE2 Induced by Silica Nanoparticles
Source: Biomedicines. 2023 Sep 25;11(10):2628. doi: 10.3390/biomedicines11102628 (PMC10604097; doi:10.3390/biomedicines11102628)
Supplement: Supplementary file 1 [file biomedicines-11-02628-s001.zip › biomedicines-2606321-supplementary.pdf]

Supplementary Figure 1

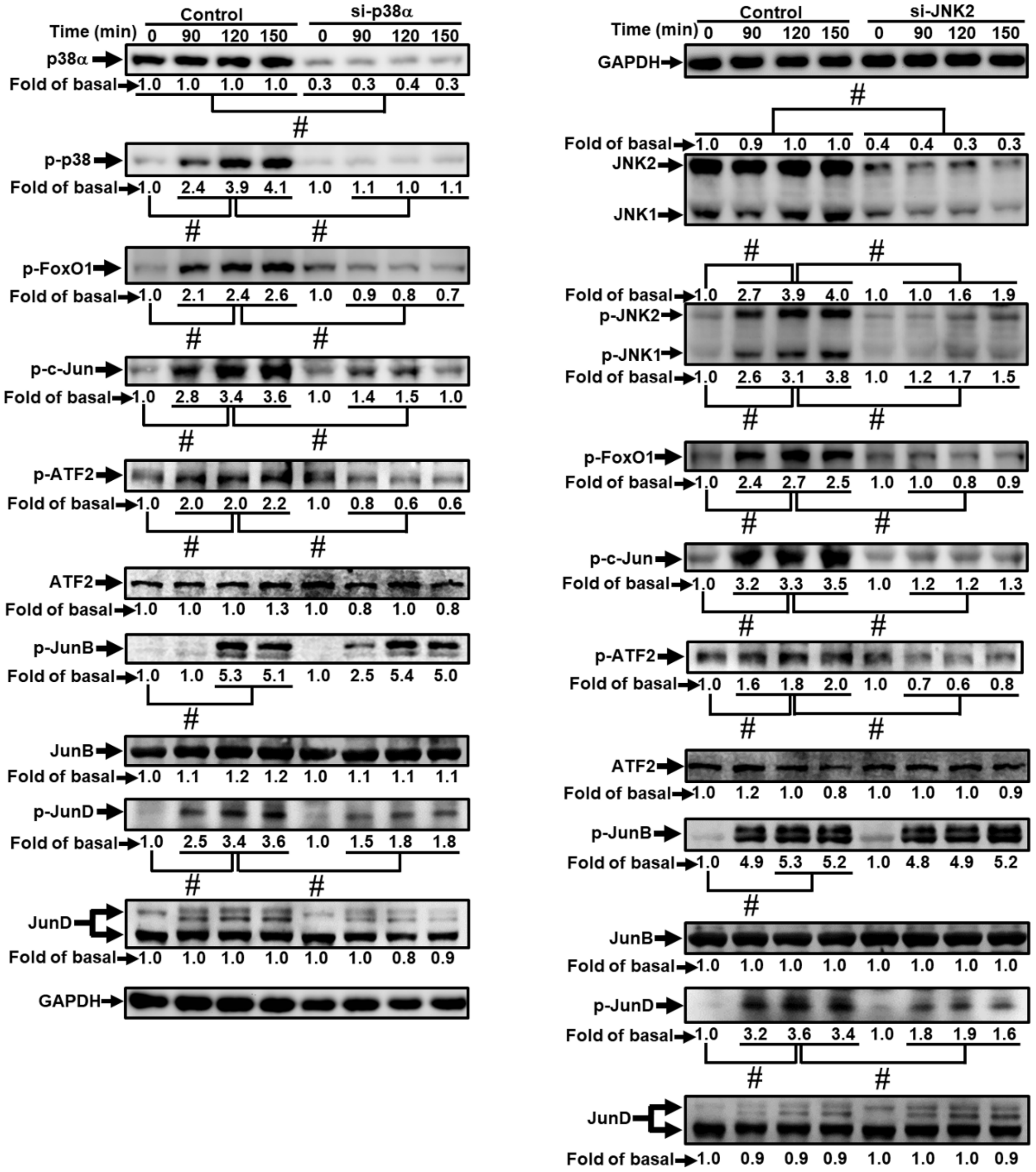

Supplementary Figure S1. In HPAEpiCs, p38 MAPK and JNK1/2 play a role in the phosphorylation of AP-1 proteins induced by SiNPs. Cells were transfected with siRNA of scrambled, (A) p38, or (B) JNK2 and then incubated with 25 μg/ml SiNPs for the indicated times. The protein levels of phospho-p38, total p38, phospho-JNK1/2, total JNK1/2, phospho-c-Jun, phospho-ATF2, total ATF2, phospho-JunB, total JunB, phospho-JunD, total JunD, and GAPDH were analyzed by Western blot. Data are expressed as the mean±S.E.M. of 3 independent experiments. #p<0.01, as compared between the two indicated groups.
